# Picosecond carrier dynamics induced by coupling of wavefunctions in a Si-nanodisk array fabricated by neutral beam etching using bio-nano-templates

**DOI:** 10.1186/1556-276X-7-587

**Published:** 2012-10-24

**Authors:** Takayuki Kiba, Yoshiya Mizushima, Makoto Igarashi, Seiji Samukawa, Akihiro Murayama

**Affiliations:** 1 Graduate School of Information Science and Technology, Hokkaido University, Kita 14, Nishi 9, Kita-ku, Sapporo, 060-0814, Japan; 2 Institute of Fluid Science, Tohoku University, 2-1-1 Katahira, Aoba-ku, Sendai, 980-8577, Japan; 3 Japan Science and Technology Agency, CREST, 5 Sanbancho, Chiyoda, Tokyo, 102-0075, Japan

**Keywords:** neutral beam etching, silicon nanostructures, bio-nano-templates, time-resolved photoluminescence, carrier dynamics

## Abstract

The picosecond carrier dynamics in a closely packed Si-nanodisk (Si-ND) array with ultrathin potential barrier fabricated by neutral beam etching using bio-nano-templates was investigated by time-resolved photoluminescence (PL). The PL decay curves were analyzed as a function of photon energy by the global fitting method. We show three spectral components with different decay times, where the systematic energy differences of the spectral peaks are clarified: 2.03 eV for the fastest decaying component with a decay time *τ* = 40 ps, 2.02 eV for *τ* = 300 ps, and 2.00 eV for *τ* = 1.6 ns. These energy separations ranging from 10 to 30 meV among the emissive states can be attributed to the coupling of wavefunctions of carriers between neighboring NDs.

## Background

Semiconductor nanostructures have been paid much attention because of their potential applications to optical devices with improved performances or novel functionalities. Quantum effects in the nanostructures are essential to realize those optical applications. The quantum effects can also affect the carrier dynamics in the nanostructures because carrier transfer or separation can be understood by a combined effect of the spatial distribution of carrier wavefunction with the energy relaxation of carriers. Therefore, the size, density, and interspacing of the nanostructure, which strongly modify the carrier wavefunction, need to be precisely controlled for the device applications. For this purpose, we have recently reported Si-nanodisk (Si-ND) arrays fabricated by neutral beam etching using bio-nano-templates [1]. This method allowed one to provide closely packed high-density Si-ND arrays. These nanostructures were difficult to prepare by means of conventional self-assembly methods using co-sputtering and annealing. In this characteristic nanostructure, wavefunctions of photoexcited carriers can spread over neighboring NDs because of the periodic alignment of the NDs with ultrathin potential barriers. This spatial overlapping of the wavefunction will result in an ultrafast carrier transfer among the NDs, which can be induced by the ultrafast energy relaxation of the carriers into NDs with lower potentials. It is important to point out that superlattice-type Si-NDs based on the ND alignment used in this study are efficient for future applications to quantum dot solar cells with high efficiencies if photoexcited electron–hole pairs are immediately separated after the excitation. In this report, we investigate the carrier dynamics induced by the lateral coupling of the carrier wavefunctions in a single layer of a two-dimensional Si-ND array by means of time-resolved photoluminescence (PL).

## Methods

The Si-ND array was fabricated by damage-free neutral beam etching using a bio-template composed of ferritin supramolecules. Details of the fabrication process are described elsewhere [1-3]. The ND thickness, diameter, and interspacing distance were designed at 8, 10, and 2 nm, respectively. Optical excitation was performed by second-harmonic fs pulses with a wavelength of 400 nm, pulse width of 150 fs, and repetition rate of 76 MHz, produced using a mode-locked Ti:sapphire laser. The time-resolved PL spectra were observed at various temperatures and excitation densities using a synchroscan streak camera. The time width of the instrumental response curve was less than 15 ps, and the time resolution of 5 ps was estimated after deconvolution with the instrumental response.

## Results and discussion

**Figure 1:**
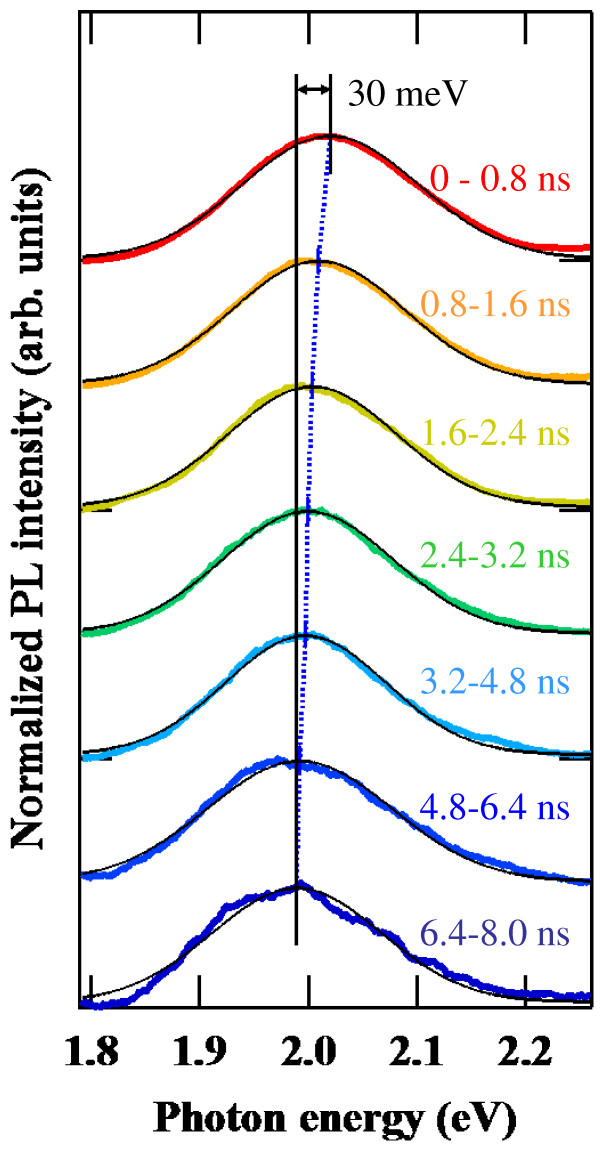
**Time-resolved PL spectra of the Si-ND array at 250 K.** Solid black lines show the fitting result with a Gaussian function.

The time-resolved PL spectra of the Si-ND array at 250 K with an excitation power of 50 mW (approximately 8.4 mJ cm^−2^) are shown in Figure 1. The PL emission band appeared at around 2.0 eV. The PL in a visible light region was characteristic in many types of Si nanostructures. The PL peak energy systematically shifts towards the lower energy side as the time elapsed, where each PL spectrum at different time regions is fitted by a single Gaussian function. From this fitting, the energy shift of the PL peak is quantitatively clarified to be about 30 meV up to 8.0 ns after the pulsed excitation. No significant time-dependent change in the spectral shape or width was observed.

The observed PL cannot be attributed to the indirect bandgap emission affected by the quantum confinement effect, which was often reported in studies using small Si nanocrystals with diameters of 2 to 3 nm [4,5], because this observed emission energy which is observed in the present NDs is remarkably higher than the value of 1.7 eV which is reported in small Si nanocrystals with an averaged diameter of 2.5 nm [4]. Moreover, lifetimes of the observed PL, which is in the range of 40 ps to 1.6 ns and will be discussed later, are much shorter than those of the microsecond scale, characteristic of the indirect bandgap recombination of carriers. A possible origin of the present PL is the emissive surface states which are weakly located around the interface of Si/SiO_2_. There are also several reports for surface-related emissions in the visible region, and it has been confirmed by PL measurements of some samples with different surface treatments [6]. It should be noted that the relatively strong PL with the very fast decay times implies the good optical qualities of these Si-NDs without defects. The other presumable interpretation is the quasi-direct gap emission. Dhara et al. have reported the PL emission with the wavelength of about 600 nm and decay times of several nanoseconds [7]. They assigned this PL to the quasi-direct bandgap emission in heavily strained Si-NCs.

The spectral linewidths of single Si nanocrystals were reported to be 100 meV or more [8,9], which were also dependent on the fabrication method and surface conditions. In our case, the size of the Si ND was precisely controlled by the diameter of the Fe core which formed in a cavity of the ferritin molecule. The size uniformity of 8% was calculated from the statistical analysis of SEM images [1]. The full width at half maximum of the PL band observed in this study is in fact 200 meV, which is a not-so-large value as compared with the above results for single nanocrystals. These considerations reveal that inhomogeneous spectral broadening and the resultant spectral diffusion due to the size distribution of Si NDs are not significant in the present study. In addition to this, the rise component of the PL decay curve was not apparently observed even in the lower energy side of the PL spectra. The absence of the rise component indicates that the time-dependent spectral shift observed in Figure 1 is not caused by the spectral diffusion, which is a time-dependent red shift of the PL energy originating from the carrier migration among high-density NDs for seeking lower potential minima.

**Figure 2:**
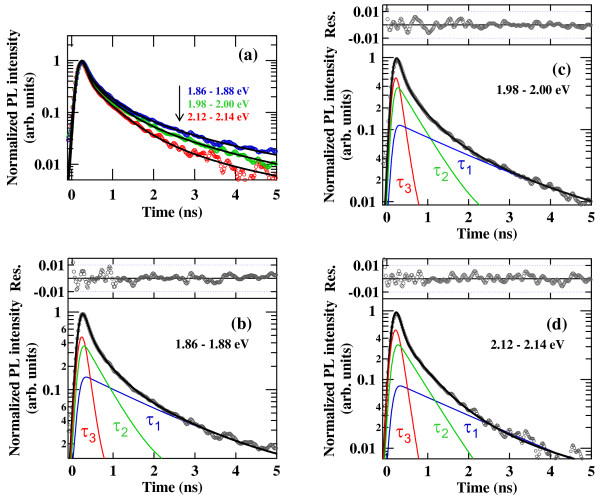
**PL time profiles and fitting results.** (**a**) PL time profiles of the Si-ND array at different energy regions. Solid lines are the result of global fitting analysis. (**b**, **c**, **d**) Typical fitting results of the PL time profile using a triple exponential function, where the PL time profile is deconvoluted with an instrumental response function. A black line shows a fitting calculation, and each decaying component is shown by a narrow line. The upper panel shows a residual difference between the experimental and simulated values.

**Figure 3:**
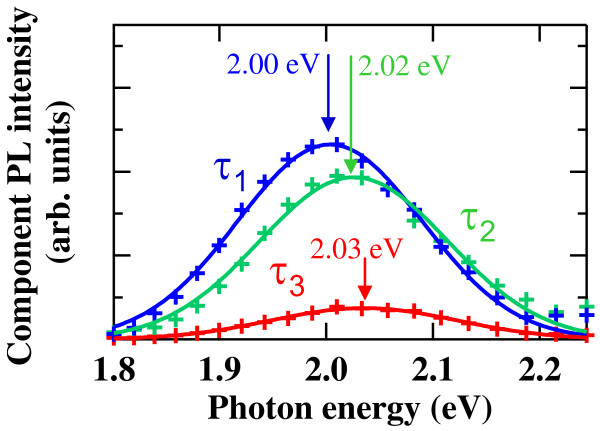
**Decay-associated PL spectra.** The spectra were obtained from the global fit analysis with linked time constants of a triple exponential function expressing the PL decay curve. Solid lines show fitting curves using Gaussian functions. A peak energy for each PL component is also indicated by an arrow.

Figure 2 shows typical PL decay curves at different PL energy regions in the Si-ND array, where the time-resolved PL spectra were divided into 20 energy regions. The time-sliced curve of PL intensity in each energy region was fitted using a triple-exponential function taking convolution of the instrumental response function into account. The temporal evolution of each PL decay could be expressed by neither single and double exponential functions nor stretched exponential function. At least, triple exponential components were required to obtain the best fit. It was also difficult to determine unique parameter sets when we increased the number of component, though we could reproduce the experimental data well. Therefore, we applied the global fitting analysis with linked decay times in order to avoid ambiguities of the fitting parameters. For this analysis, all decay curves were fitted simultaneously using three universal decay time constants, and we obtained individual amplitudes of three decaying components for each energy region. Then, we could reproduce the PL component spectra associated to the decay times by plotting the time-integrated intensities as a function of the photon energy for the three decaying components. The result is shown in Figure 3, where we have identified the three PL decaying components with different time constants *τ*_1_ = 1.6 ns, *τ*_2_ = 300 ps, and *τ*_3_ = 40 ps, respectively.

We shall provide a possible explanation for the existence of the three PL components with different decay times. We assigned these three decaying components to three different types of emissive sites in the Si-ND array from the disk-density and excitation-power dependences of the PL decay time and intensity [10]. First, the emission component with the slowest decay time *τ*_1_ on the order of 1 ns was interpreted by electron–hole pairs localized at individual NDs because this PL component was observed to be dominant when we excited lower-density NDs with the disk interspacing larger than 40 nm. This trend was independent of the excitation density, which suggested the absence of the excited state. In contrast, the two faster PL components with *τ*_2_ and *τ*_3_ appeared only when we excited the high-density ND array with relatively high excitation powers. Therefore, these faster emissions were understood by the recombination of electron–hole pairs which are not strongly localized in each ND, where each wavefunction of the carrier spreads over neighboring NDs. The fastest PL component with *τ*_3_ was particularly attributed to the recombination involving the electron transfer among the NDs which are induced by the periodic regular alignment of the ND separated by ultrathin potential barriers. In other words, the fastest PL was quenched by the electron transfer.

As can be seen in Figure 3, the decay-associated component PL spectra show the different energy peaks. The peak energy of the component PL spectrum associated to the slowest decaying component (*I*_1_ with *τ*_1_) is meaningfully lower than those of the faster two components (*I*_2_ with *τ*_2_, and *I*_3_ with *τ*_3_). The energy difference between *I*_1_ and *I*_2_ is 20 meV, and that between *I*_1_ and *I*_3_ is 30 meV, which are quantitatively obtained from the fittings for the decay-associated component PL spectra with Gaussian functions. These energy differences mean the energy separations among the energy levels of individual emissive electronic states discussed above. The lowest energy state associated to the slowest PL component (*I*_1_ with *τ*_1_) originates from the electron–hole pairs that are weakly localized in surface states in individual NDs, which corresponds to the ground state in the present high-density ND system, and the wavefunctions of carriers are localized within one ND. Higher-energy levels with faster decay times (*I*_2_ with *τ*_2_, and *I*_3_ with *τ*_3_) are excited states in the ND system, and the coupling of carrier wavefunctions among NDs can form these excited states. Therefore, we consider that the obtained values of 20 to 30 meV of the energy differences among the decay-associated PL components can be explained by the energy differences between the ground state and the excited states in the present coupled Si-ND array structure. We have also confirmed this result from the temperature dependences of the decay-associated PL intensities. The intensities of these three spectral components were measured with varying temperatures, and the results were fitted by calculations taking activation energies between each energy level as well as the barrier level into account. The differences of the activation energy also indicate the energy differences of the lower two states, and the obtained value is 55 meV between the slowest component (*τ*_1_) and the middle component (*τ*_2_), which agrees with the results obtained from the global fitting method. Further study to test the energetic structure experimentally elucidated here in the Si-ND system is needed using three-dimensional calculations of the carrier wavefunctions based on the actual ND array structure.

## Conclusions

The carrier dynamics in the high-density Si-ND array fabricated by neutral beam etching using bio-nano-templates was studied by time-resolved PL, where the PL decay curves were analyzed at various photon energies by the global fitting method. The obtained decay-associated spectral components show clear differences of the peak energy of 20 to 30 meV between the slowest component (*τ*_1_) and the faster two components (*τ*_2_, *τ*_3_). These energy differences for the emissive states can be attributed to the different coupling states of the carrier wavefunctions in the present high-density ND system. The ultrafast carrier dynamics elucidated from the time-resolved PL analysis demonstrates that high-density semiconductor nanostructures precisely designed and fabricated by neutral beam etching using bio-nano-templates are promising for optical applications based on quantum effects such as quantum dot lasing and photovoltaics.

## Abbreviations

ND: Nanodisk; PL: Photoluminescence.

## Competing interests

The authors declare that they have no competing interests.

## Authors’ contributions

TK and AM conceived the spectroscopic study, participated in its design and coordination, and drafted the manuscript. TK and YM carried out the time-resolved PL measurement and analyzed the data. MI and SS conceived the fabrication process and participated in its design and coordination. MI fabricated the Si-ND array sample. All authors read and approved the final manuscript.

## References

[B1] Huang CH Wang X-Y Igarashi M Murayama A Okada Y Yamashita I Samukawa S Optical absorption characteristic of highly ordered and dense two-dimensional array of silicon nanodiscs Nanotechnology 2011 22 105301 10.1088/0957-4484/22/10/105301 21289397

[B2] Huang C-H Igarashi M Woné M Uraoka Y Fuyuki T Takeguchi M Yamashita I Samukawa S Two-dimensional Si-nanodisk array fabricated using bio-nano-process and neutral beam etching for realistic quantum effect devices Jpn J Appl Phys 2009 48 04C187 10.1143/JJAP.48.04C187

[B3] Kubota T Hashimoto T Takeguchi M Nishioka K Uraoka Y Fuyuki T Yamashita I Samukawa S Coulomb-staircase observed in silicon-nanodisk structures fabricated by low-energy chlorine neutral beams J Appl Phys 2007 101 124301 124309 10.1063/1.2747226

[B4] de Boer WDAM Timmerman D Dohnalova K Yassievich IN Zhang H Buma WJ Gregorkiewicz T Red spectral shift and enhanced quantum efficiency in phonon-free photoluminescence from silicon nanocrystals Nat Nanotechnol 2010 5 878 884 10.1038/nnano.2010.236 21113157

[B5] Conibeer G Green M Corkish R Cho Y Cho E-C Jiang C-W Fangsuwannarak T Pink E Huang Y Puzzer T Trupke T Richards B Shalav A Lin K-l Silicon nanostructures for third generation photovoltaic solar cells Thin Solid Films 2006 511-512 654

[B6] Ray M Hossain SM Robert FK Banerjee K Ghosh S Free standing luminescent silicon quantum dots: evidence of quantum confinement and defect related transitions Nanotechnology 2010 21 505602 10.1088/0957-4484/21/50/505602 21098931

[B7] Dhara S Giri P Size-dependent visible absorption and fast photoluminescence decay dynamics from freestanding strained silicon nanocrystals Nanoscale Res Lett 2011 6 320 10.1186/1556-276X-6-320 21711845 PMC3211408

[B8] Kůsová K Cibulka O Dohnalová K Pelant I Valenta J Fučíková A Zídek K Lang J Englich J Matejka P Štĕpánek P Bakardjieva S Brightly luminescent organically capped silicon nanocrystals fabricated at room temperature and atmospheric pressure ACS Nano 2010 4 4495 10.1021/nn1005182 20690596

[B9] Martin J Cichos F Huisken F von Borczyskowski C Electron–phonon coupling and localization of excitons in single silicon nanocrystals Nano Lett 2008 8 656 660 10.1021/nl0731163 18197721

[B10] Kiba T Mizushima Y Igarashi M Huang C-H Samukawa S Murayama A Picosecond transient photoluminescence in high-density Si-nanodisk arrays fabricated using bio-nano-templates Appl Phys Lett 2012 100 053117 10.1063/1.3681793 PMC3499154 23095286

